# Supplementary material for: Metal 3D printing technology for functional integration of catalytic system
Source: Nat Commun. 2020 Aug 14;11:4098. doi: 10.1038/s41467-020-17941-8 (PMC7428005; doi:10.1038/s41467-020-17941-8)
Supplement: Supplementary file 1 — Supplementary Information [file 41467_2020_17941_MOESM1_ESM.pdf]

Supplementary Information for

Metal 3D Printing Technology for Functional Integration of Catalytic System

Wei et al.

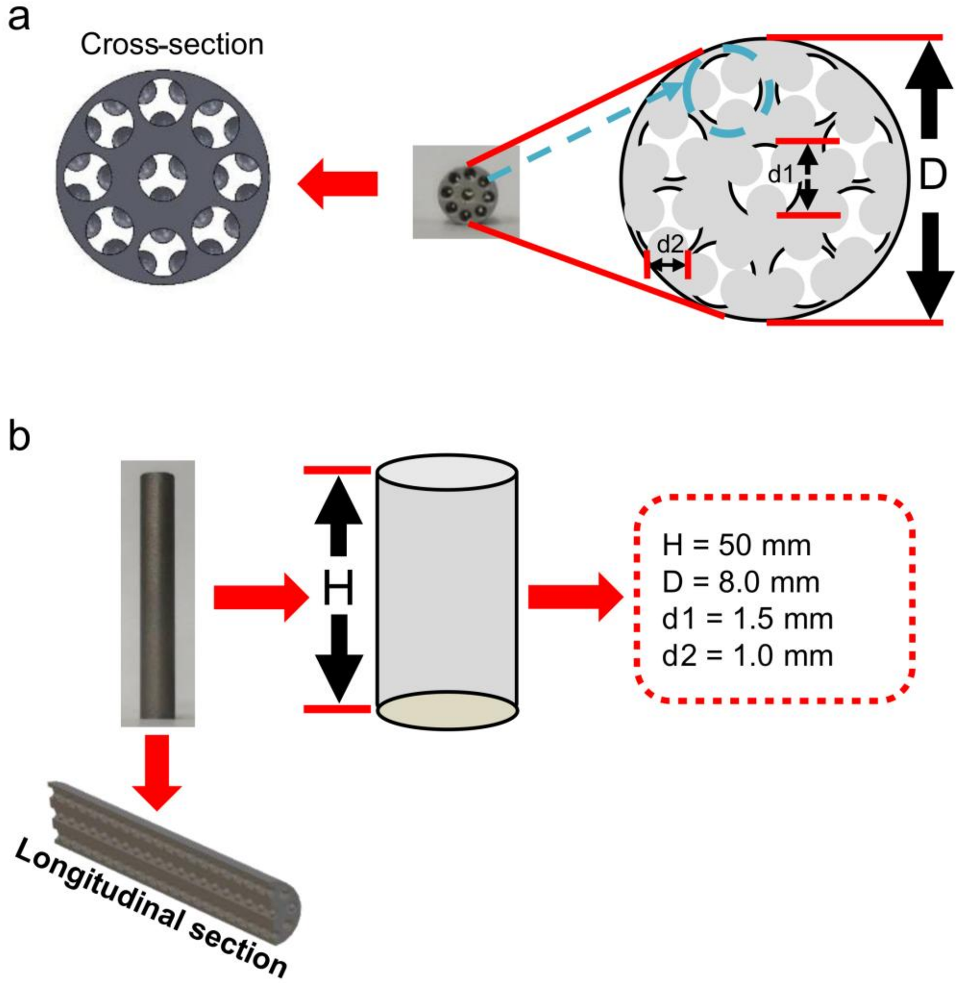

Supplementary Figure 1 | Structural parameters of M (M = Fe, Co, Ni)-SCR. (**a**) Cross-section of the M-SCR; (**b**) detailed parameters of the M-SCR. To increase the inner surface area, the M-SCR was designed with 9 channels. Each channel consisted of 3 rows of small semicircular spheres, and one row had 20 semicircular spheres.

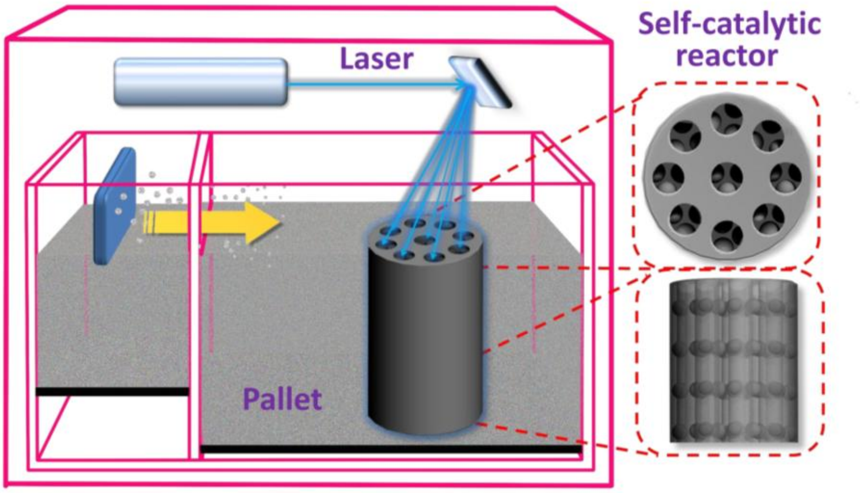

Supplementary Figure 2 | Schematic diagram for SCR fabrication via selective laser sintering.

Supplementary Table 1 | The elemental composition for Fe-Powder.

Sample	Metal contents (wt%)				
	Fe	Ni	Co	Mo	Ti
Fe-Powder	65.6	17.8	10.1	5.4	1.1

The elemental composition of Fe-Powder was obtained by XRF analysis.

Supplementary Table 2 | The elemental composition for Co-Powder.

Sample	Metal contents (wt%)				
	Co	Cr	Mo	W	Others
Co-Powder	61.0	27.0	6.0	5.0	1.0

The elemental composition of Co-Powder was obtained by XRF analysis.

Supplementary Table 3 | The elemental composition for Ni-Powder.

Sample	Metal contents (wt%)					
	Ni	Fe	Cr	Nb	Mo	Others
Ni-Powder	53.6	18.6	17.7	5.2	3.2	1.7

The elemental composition of Ni-Powder was obtained by XRF analysis.

Supplementary Table 4 | BET area for Fe-Powder and Fe-SCR before and after the pretreatments.[a]

Sample	BET area ($m^2\ g^{-1}$)[b]
Fe-Powder	12
Fe-Powder-*Calcined*	12
Fe-Powder-*Reduced*	13
Fe-Powder-*Spent*	13
Fe-SCR	1.6
Fe-SCR-*Calcined*	2.6
Fe-SCR-*Reduced*	2.7
Fe-SCR-*Spent*	3.0

(**a**) SiO_2 pellet was used as an inner standard for the BET analysis. (**b**) The SCR samples were cut into small pieces, and the weight of each piece was about 0.20~0.25 g for the BET analysis.

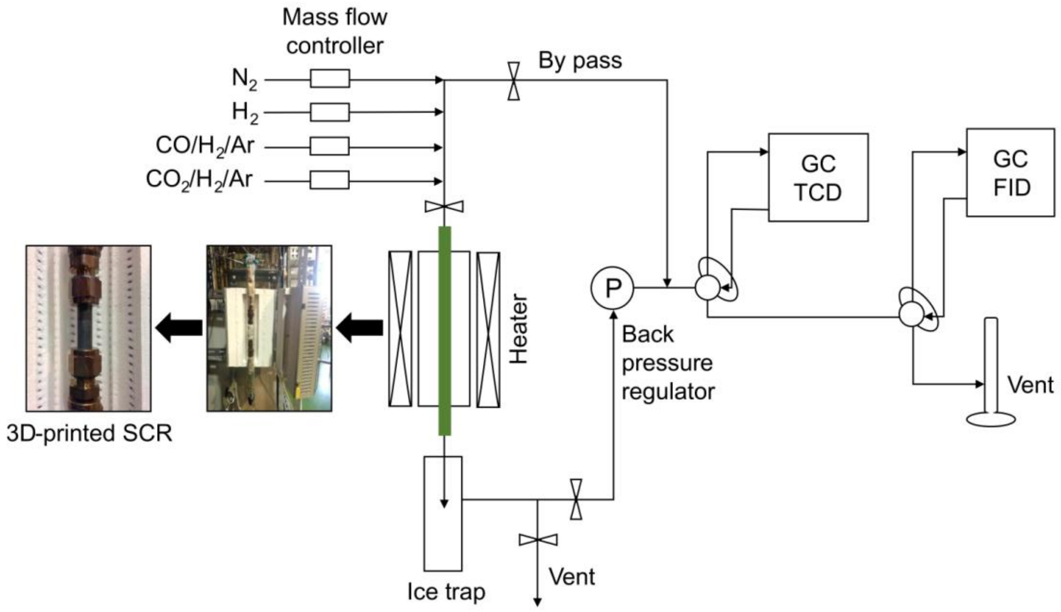

Supplementary Figure 3 | Schematic of reaction devices for FT synthesis and CO_2 hydrogenation. The Fe-SCR or Co-SCR was directly linked in the reaction apparatus. Mixture gas, such as CO/H_2 or CO_2/H_2, was fed in this catalytic system, with Ar as an inner standard. Then, the system was raised to the desired temperature and pressure, to start the reaction. The effluents were analyzed by two online gas chromatographies (Shimadzu GC-8A with thermal conductivity detector (TCD); Shimadzu GC-14B with flame ionization detector (FID)). The ice trap was used to capture liquid hydrocarbons. The liquid hydrocarbons were analyzed by an off-line gas chromatography (Shimadzu GC-2014 with FID).

Supplementary Table 5 | FT performance over the T_{316}, Fe-Powder and Fe-SCR.[a]

Sample	CO Conv. (%)	CO_2 Selectivity (%)[b]	Hydrocarbon Selectivity (%)[c]			C_{ole}/C_n[d]	C_{iso}/C_n[e]
			CH_4	C_2-C_4	C_{5+}		
T_{316}	0	/	/	/	/	/	/
Fe-Powder	74	20	36	35	29	0.1	0.2
Fe-SCR	69	20	21	34	45	0.1	0.3

(**a**) Reaction conditions: T = 573 K; P = 1.0 MPa; H_2/CO = 2.0; flow rate, 20 ml min^{-1}; time on stream, 10 h. (**b**) The calculation of CO_2 selectivity based on C-moles of the CO_2 formation with respect to the C-moles of the converted CO. (**c**) The hydrocarbon selectivity was normalized with the exception of CO_2.[1,2] The calculation based on the C-moles of a product with respect to the total C-moles in the hydrocarbon mixture. (**d**) C_{ole}/C_n represents the ratio of olefins to n-paraffins in C_{2-20}. (**e**) C_{iso}/C_n represents the ratio of isoparaffins to n-paraffins in C_{4-20}.

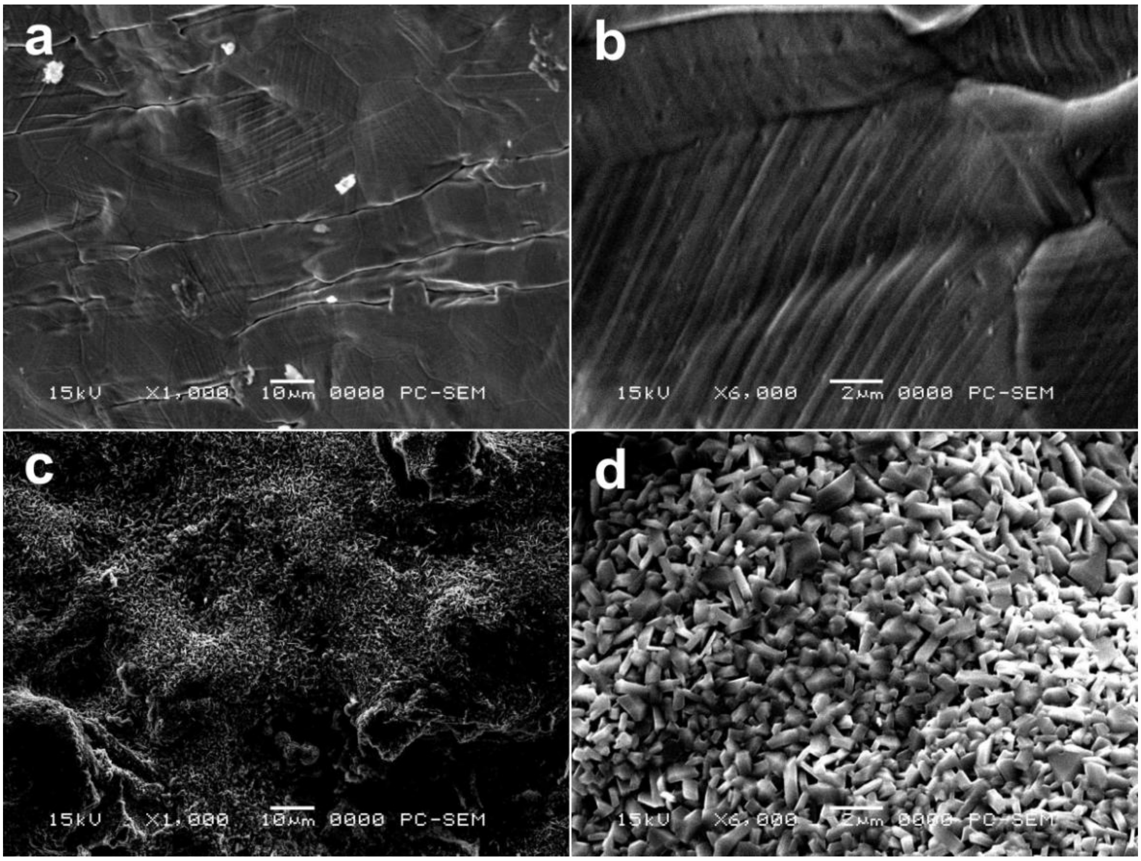

Supplementary Figure 4 | SEM images for inner morphology of the T_{316} and Fe-SCR. (**a**) SEM image for the T_{316}; (**b**) high-magnification SEM image for the T_{316}; (**c**) SEM image for the Fe-SCR; (**d**) high-magnification SEM image for the Fe-SCR.

Supplementary Table 6 | Linear velocity of syngas in the T_{316}, Fe-Powder and Fe-SCR.[a]

Sample	Inner surface (10^{-3} m^2)	Channel volume (10^{-6} m^3)	Linear velocity[c] (10^{-4} m s^{-1})
T_{316}	1.2	2.2	2.8
Fe-Powder	22[b]	/	0.2
Fe-SCR	3.9	0.6	0.8

(**a**) Inner surface and channel volume were obtained according to the reactor size and CAD calculation. (**b**) The surface of Fe-Powder was calculated based on the average particle size. (**c**) The linear velocity was calculated based on the equation of $V_{\text{linear}} = F_{\text{CO+H2}} / S_{\text{cat.}}$ (V_{linear}, $F_{\text{CO+H2}}$ and $S_{\text{cat.}}$ represent linear velocity of syngas, flow rate of syngas and catalyst surface, respectively). Syngas conditions: temperature, 573 K; pressure, 1.0 MPa; flow rate, 20 ml min^{-1}.

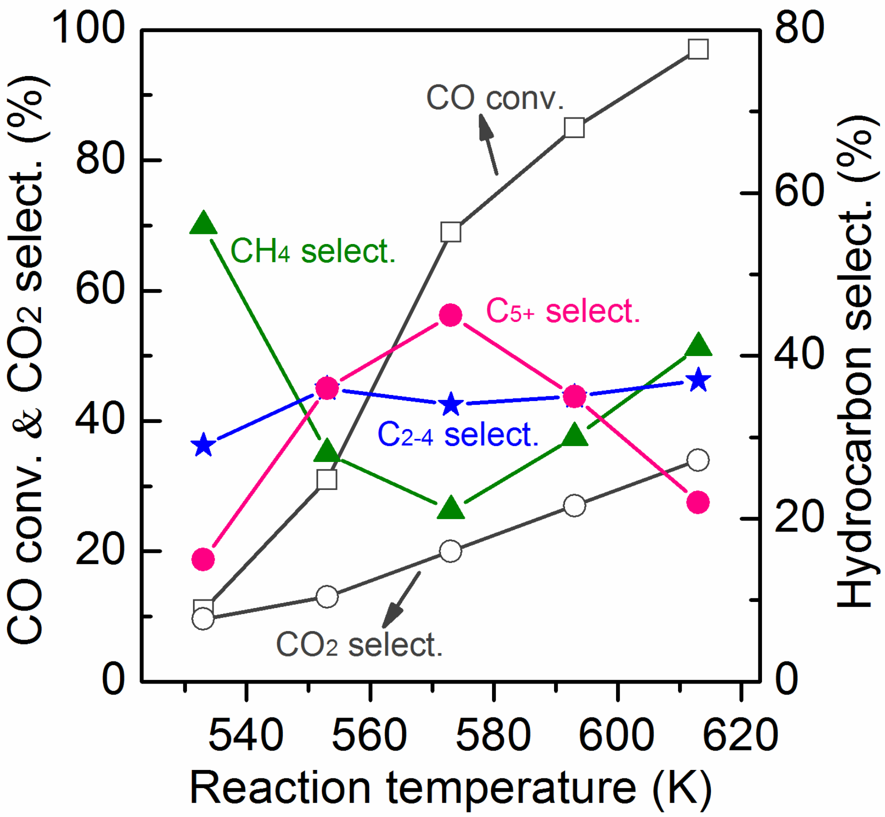

Supplementary Figure 5 | FT performance over the Fe-SCR at different reaction temperatures. Reaction conditions: P = 1.0 MPa; H_2/CO = 2.0; flow rate, 20 ml min^{-1}; time on stream, 10 h.

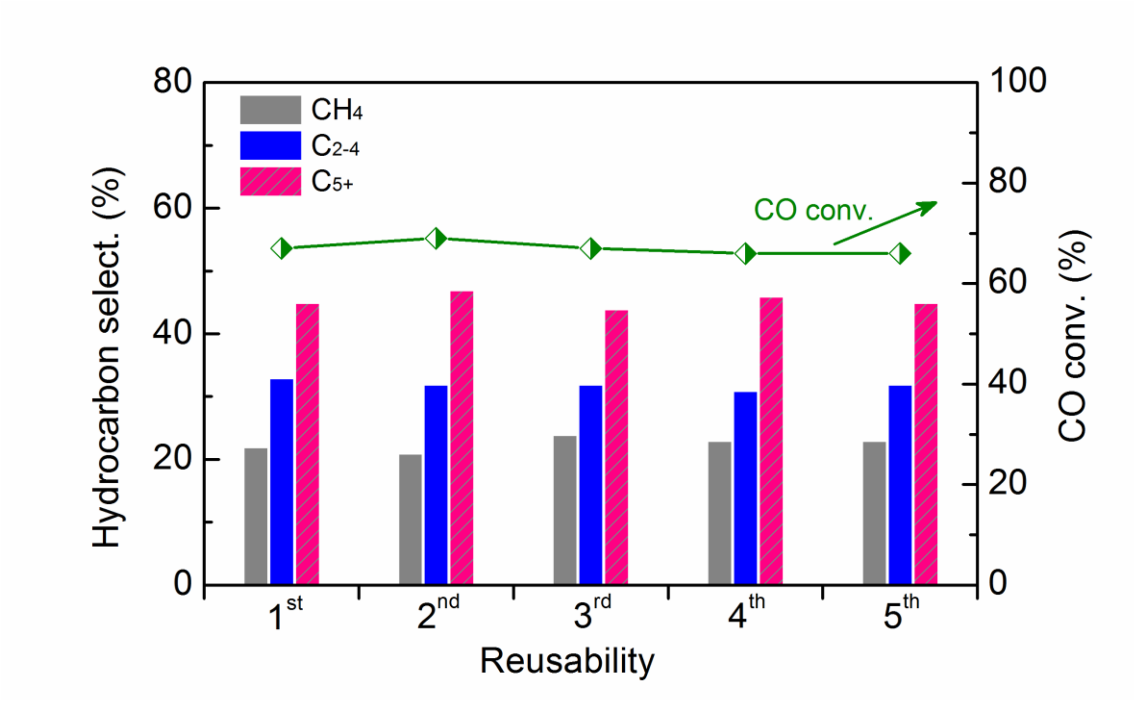

Supplementary Figure 6 | Reusability of the Fe-SCR. We reused the Fe-SCR for 5 times. The results exhibited that the CO conversion and the product distribution were almost the same, indicating a high reusability. The reaction conditions were the same with those of the Supplementary Table 5.

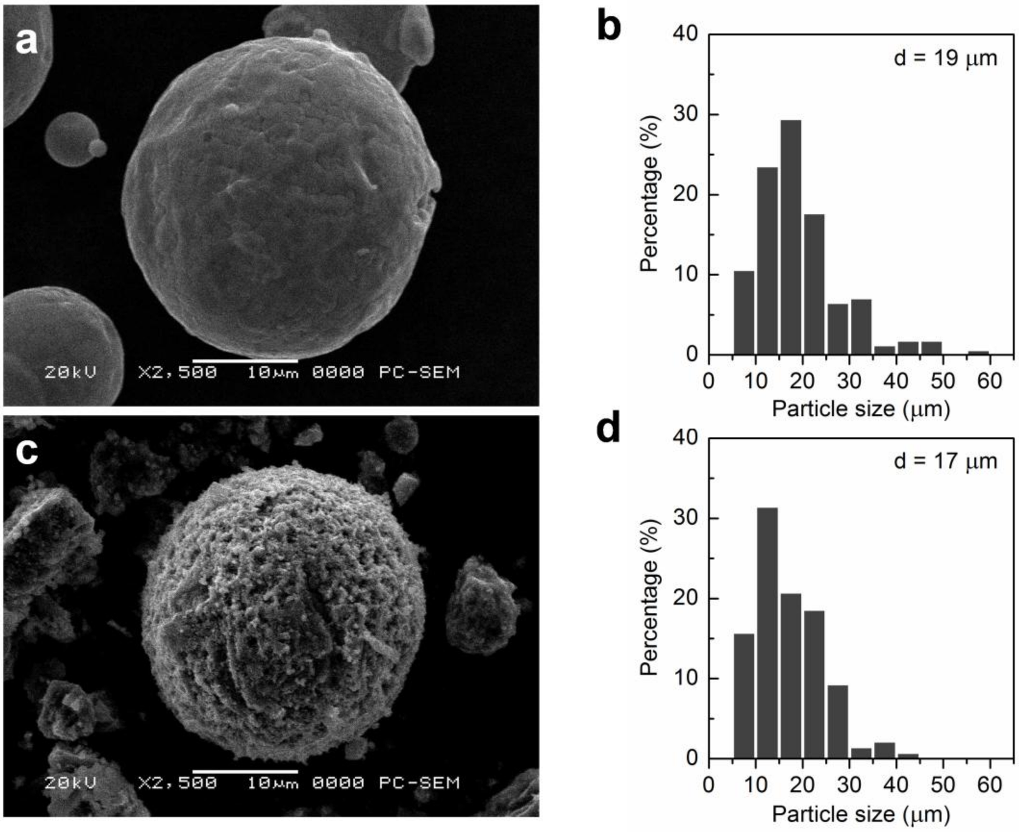

Supplementary Figure 7 | Surface morphology and particle size distribution for the Fe-Powder and Fe-Powder-*Calcined*. (**a**) SEM image for the Fe-Powder; (**b**) particle size distribution for the Fe-Powder; (**c**) SEM image for the Fe-Powder-*Calcined*; (**d**) particle size distribution for the Fe-Powder-*Calcined*. The average particle sizes of Fe-Powder and Fe-Powder-*Calcined* were about 18 μm.

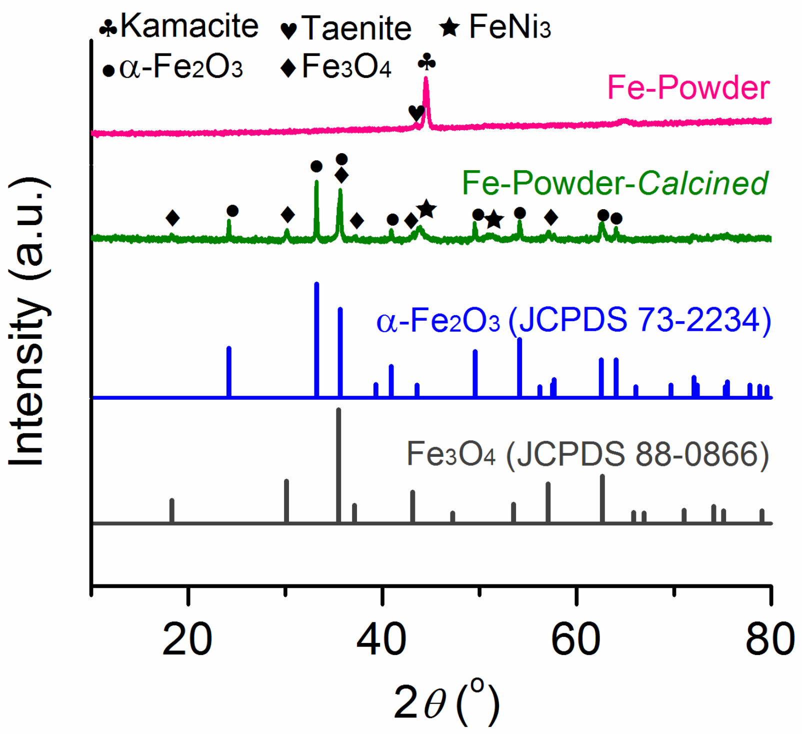

Supplementary Figure 8 | XRD patterns for the Fe-Powder and Fe-Powder-*Calcined*. Kamacite and taenite alloys were the main components for Fe-Powder. After the calcination at 873 K, the kamacite and taenite alloys were further transformed into α-Fe_2O_3 and Fe_3O_4 over the Fe-Powder-*Calcined*.

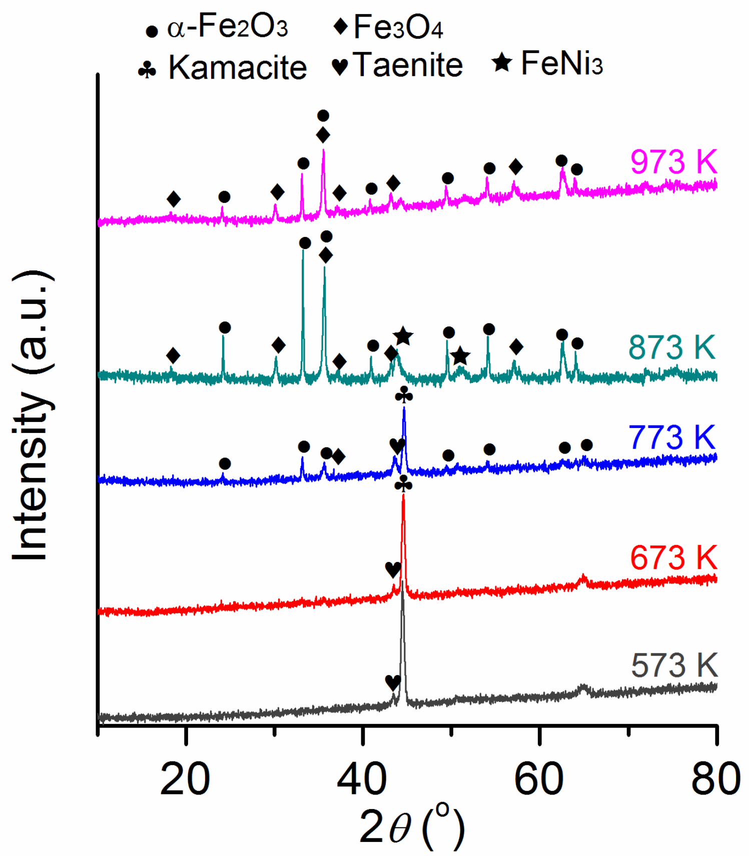

Supplementary Figure 9 | XRD patterns for the Fe-Powder-*Calcined* with different calcination temperatures. To further confirm the influence of calcination, we calcined the Fe-Powder with different temperatures. The α-Fe_2O_3 and Fe_3O_4 were formed from the temperature of 773 K. The Fe-Powder remained kamacite and taenite alloys, when the temperature was lower than 673 K.

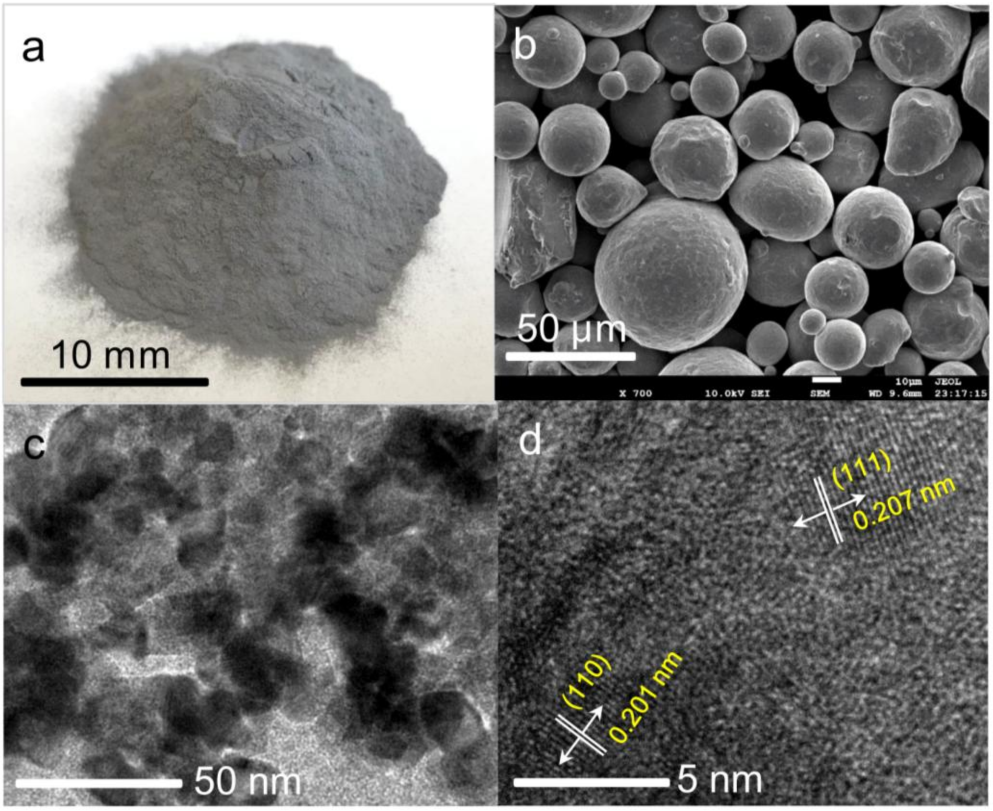

Supplementary Figure 10 | SEM and TEM images for the Fe-Powder. (**a**) A pile of Fe-Powder, (**b**) SEM image, (**c**) TEM image, (**d**) high-magnification TEM image. The values of 0.201 nm and 0.207 nm should correspond to the lattice planes of kamacite (110) and taenite (111), respectively.[3,4]

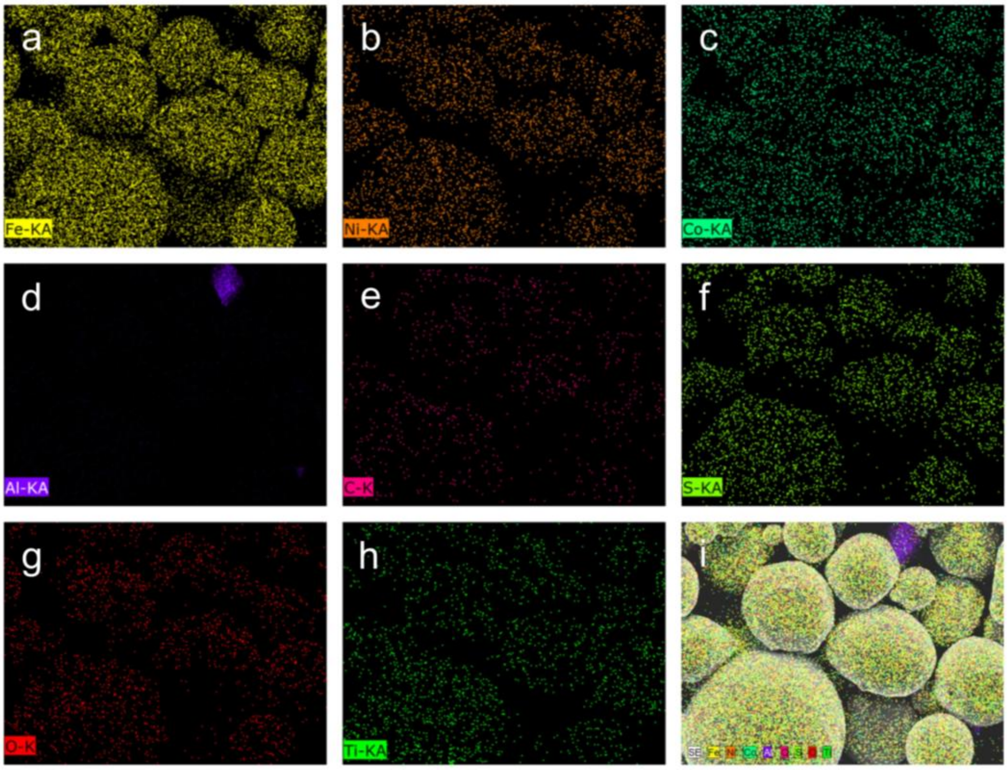

Supplementary Figure 11 | Elemental mapping of the Fe-Powder. Elemental composition: (**a**) Fe, (**b**) Ni, (**c**) Co, (**d**) Al, (**e**) C, (**f**) S, (**g**) O, (**h**) Ti, (**i**) merged image. To confirm the presence of carbon in the Fe-Powder, the sample was first coated by a aluminum foil and pasted on a conductive adhesive. Using this way, the detected carbon from conductive adhesive can be eliminated. The presence of aluminum should be attributed to the substrate of aluminum foil.

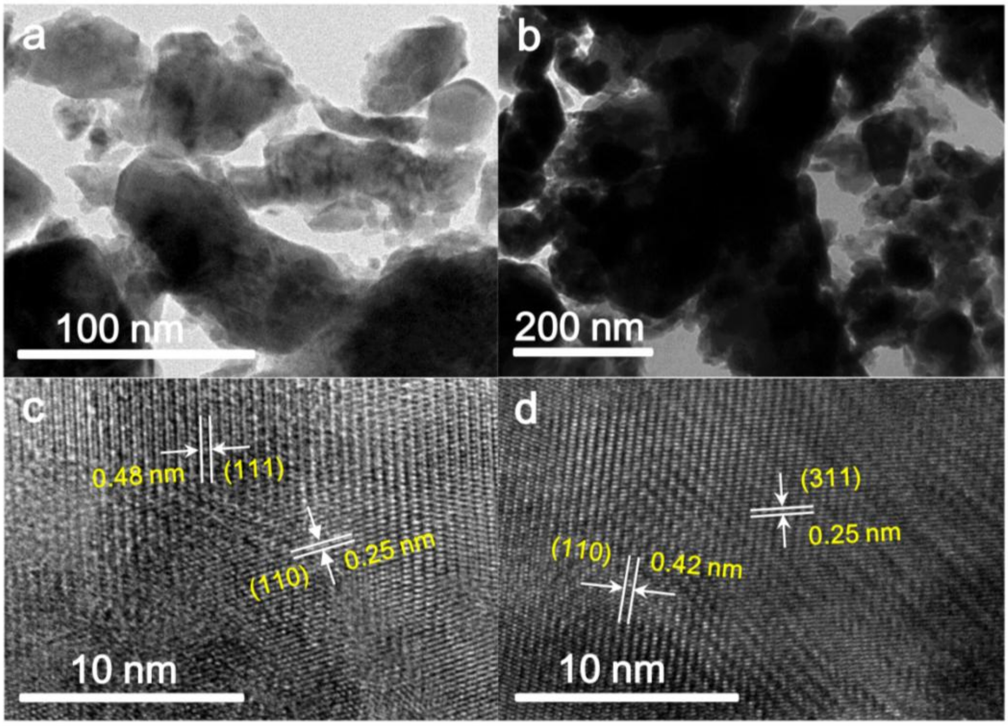

Supplementary Figure 12 | TEM images for the Fe-Powder-*Calcined* and Fe-Powder-*Spent*. (**a**) TEM image of the Fe-Powder-*Calcined*. (**b**) TEM image of the Fe-Powder-*Spent*. (**c**) High-magnification TEM image of the Fe-Powder-*Calcined*. The values of 0.25 nm and 0.48 nm should correspond to the lattice planes of α-Fe_2O_3 (110) and Fe_3O_4 (111), respectively.[5,6] (**d**) High-magnification TEM image of the Fe-Powder-*Spent*. The lattice spacings of 0.25 nm and 0.42 nm were in good agreement with those of Fe_3O_4 (311) and Fe_5C_2 (110), respectively.[5]

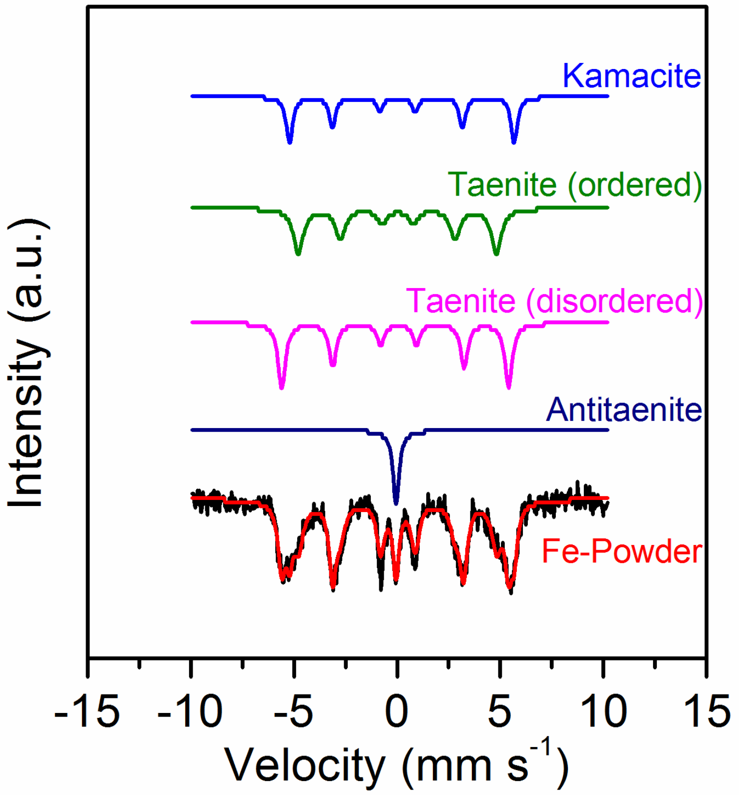

Supplementary Figure 13 | ^{57}Fe Mössbauer spectra of the Fe-Powder.[7] FeNi alloys were the main components in the Fe-Powder.

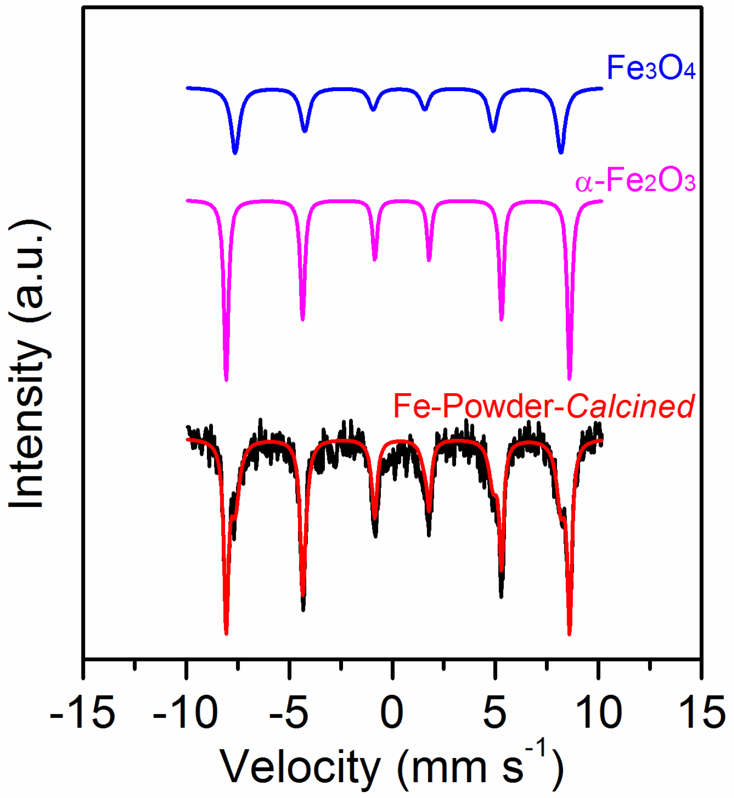

Supplementary Figure 14 | ^{57}Fe Mössbauer spectra of the Fe-Powder-*Calcined*.[8,9] After the calcination process, the phases of FeNi alloys were transformed into α-Fe_2O_3 and Fe_3O_4.

Supplementary Table 7 | ^{57}Fe Mössbauer parameters for the Fe-Powder, Fe-Powder-*Calcined*, and Fe-Powder-*Spent*.

Sample	Sub-spectrum	IS (mm/s)[a]	QS (mm/s)[b]	Hhf (kOe)[c]	Area (%)[d]
Fe-Powder	Kamacite	0.12	0.21	337	22.1
	Taenite (ordered)	0.02	-0.01	297	33.3
	Taenite (disordered)	-0.01	-0.15	340	36.1
	Antitaenite	-0.04	-	-	8.5
Fe-Powder-*Calcined*	α-Fe_2O_3	0.37	-0.2	516	58.9
	Fe_3O_4	0.29	-0.04	489	41.1
Fe-Powder-*Spent*	Fe_3O_4(A)	0.30	0.03	490	5.1
	Fe_3O_4(B)	0.66	-0.01	460	9.0
	χ-Fe_5C_2(I)	0.17	0.1	187	27.8
	χ-Fe_5C_2(II)	0.24	0.09	213	12.7
	χ-Fe_5C_2(III)	0.21	0.10	109	9.5
	α-Fe	0.00	0.00	336	31.2
	Fe^{3+}	0.24	0.96	-	4.7

(**a**) Isomer shift, (**b**) quadrupole splitting, (**c**) internal hyperfine magnetic field, (**d**) fitted peak area.

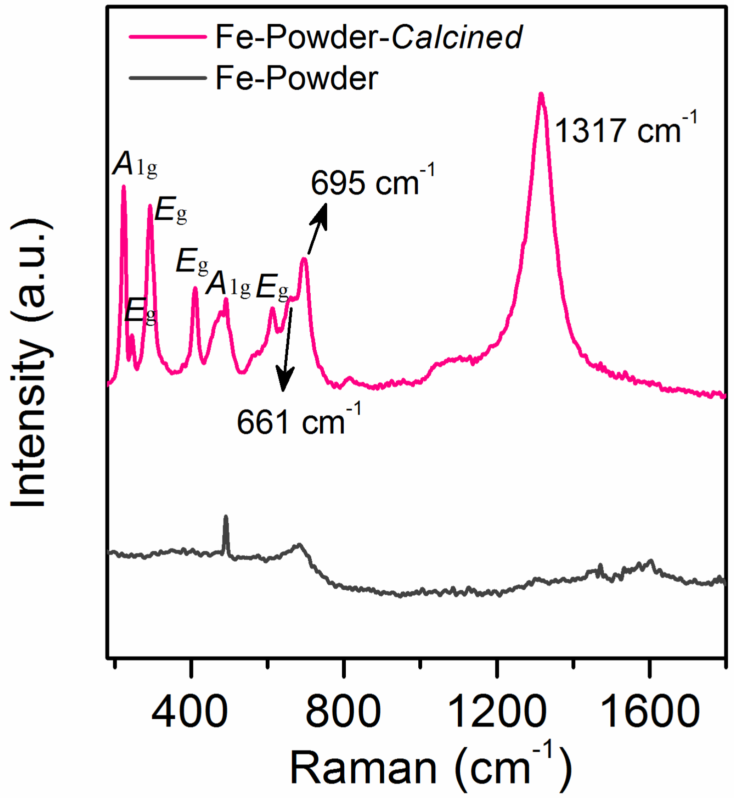

Supplementary Figure 15 | Raman spectra for the Fe-Powder and Fe-Powder-*Calcined*.[10-13] For the Fe-Powder-*Calcined*, the peaks at 224, 247, 293, 411, 493, 613 cm^{-1} were in agreement with the modes of A_{1g} (224 and 493 cm^{-1}) and E_g (247, 293, 411 and 613) of α-Fe_2O_3. The strong peak at 1317 cm^{-1} corresponded to a two-magnon scattering arising from antiferromagnetic structure of α-Fe_2O_3. In addition, the characteristic peak at 661 cm^{-1} was assigned to Fe_3O_4, and the peak at 695 cm^{-1} should be due to the A_g vibration mode of $NiFe_2O_4$. For the Fe-Powder, we did not observe any characteristic peaks for these Fe oxides. Moreover, in combination with the analyses of XRD, TEM, and ^{57}Fe Mössbauer spectra, we further confirmed that the kamacite and taenite were mainly transformed into the α-Fe_2O_3 and Fe_3O_4 in the Fe-Powder-*Calcined*.

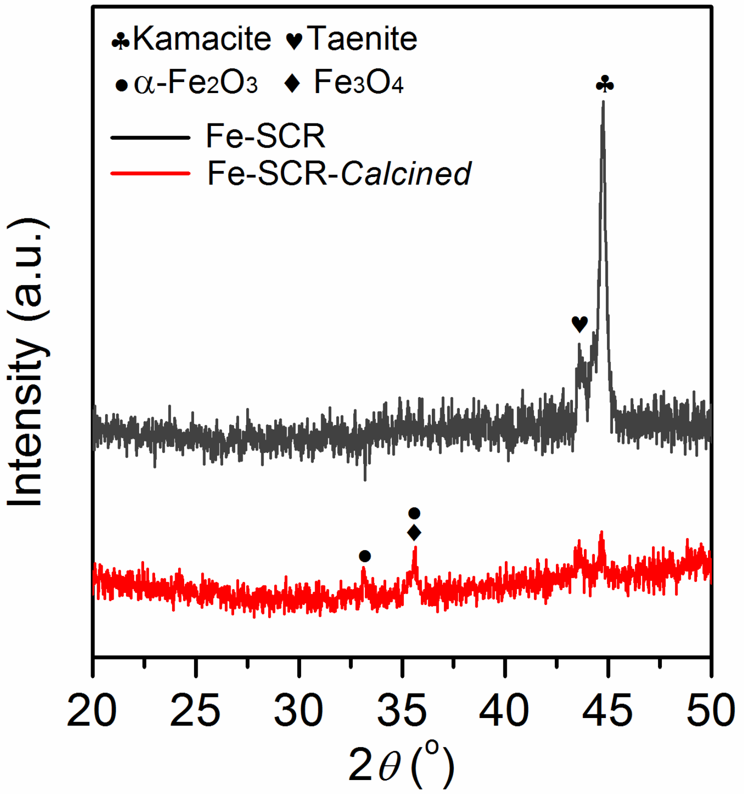

Supplementary Figure 16 | XRD patterns for the Fe-SCR and Fe-SCR-*Calcined*.

Supplementary Table 8 | EDS analysis for the Fe-Powder and Fe-SCR before and after the calcination.

Sample	Metal and non-metal contents (wt%)					
	C	O	Fe	Ni	Co	Others
Fe-Powder	0.7	0.8	66.7	17.3	8.7	5.8
Fe-SCR	3.4	1.1	65.7	16.0	8.9	4.9
Fe-Powder-*Calcined*	0.9	28.9	56.8	5.0	4.3	4.1
Fe-SCR-*Calcined*	3.2	27.2	53.0	8.6	4.1	3.9

The EDS analysis disclosed that the Fe-Powder and Fe-SCR possessed similar element distribution. The element distribution of Fe-Powder-*Calcined* was almost the same as that of the Fe-SCR-*Calcined*.

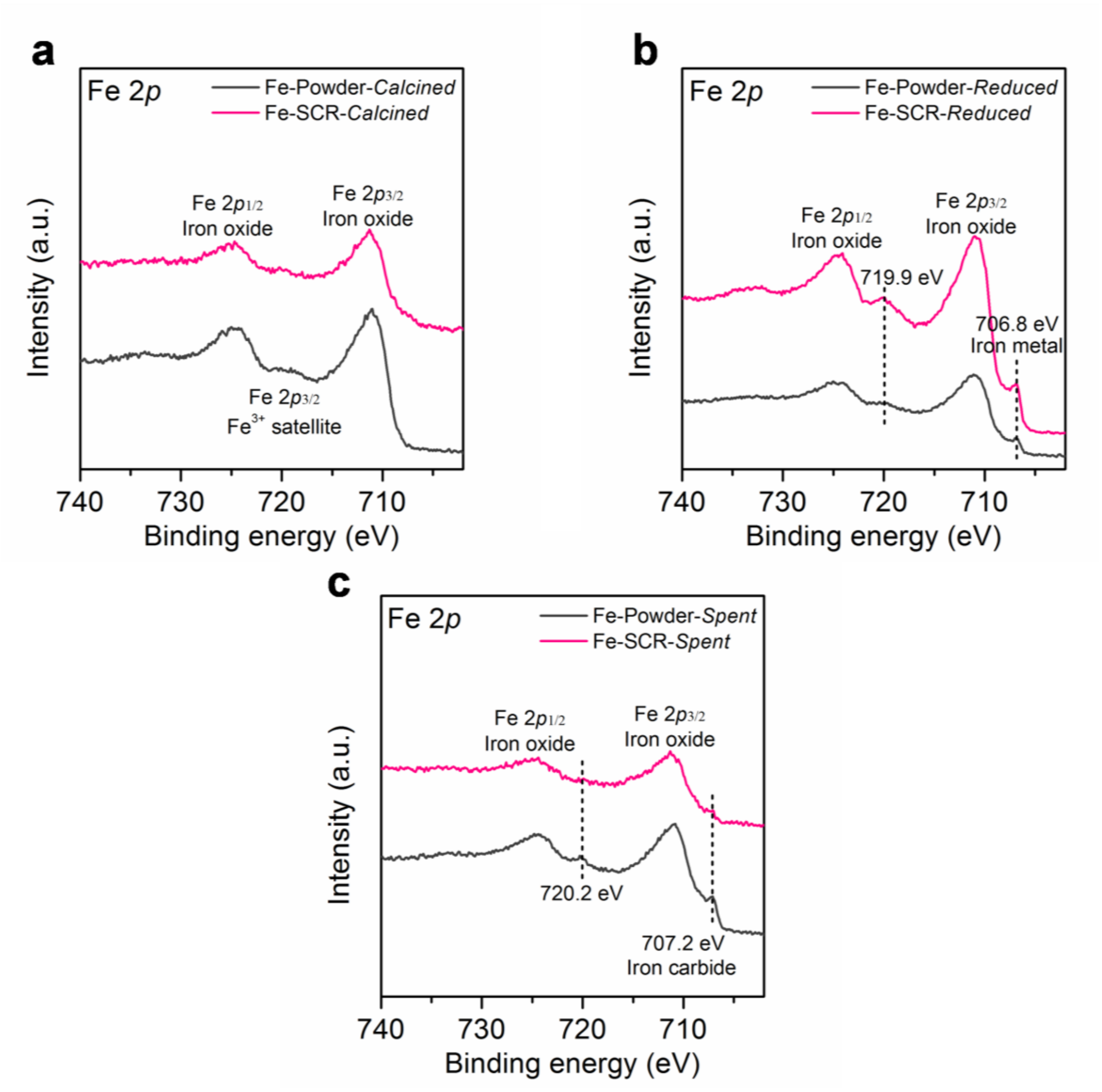

Supplementary Figure 17 | XPS spectra in Fe 2*p* region for the Fe-Powder and Fe-SCR after the pretreatments and FT reaction.[14-17] (**a**) Fe 2*p* region for the Fe-Powder-*Calcined* and Fe-SCR-*Calcined*. The Fe 2*p* peaks at 711.2 and 724.6 eV were due to $2p_{3/2}$ and $2p_{1/2}$ for iron oxide, respectively. In general, Fe_3O_4 does not show satellite in Fe 2*p* region. The satellite at 719.4 eV should be attributed to the α-Fe_2O_3. The Fe 2*p* region with the weak satellites indicates a coexistence of α-Fe_2O_3 and Fe_3O_4. (**b**) Fe 2*p* region for the Fe-Powder-*Reduced* and Fe-SCR-*Reduced*. The peak at 706.8 eV was assigned to $2p_{3/2}$ of iron metal. The peak at 719.9 eV should be due to two overlapping components: the satellite $2p_{3/2}$ of α-Fe_2O_3 and $2p_{1/2}$ of iron metal. (**c**) Fe 2*p* region for the Fe-Powder-*Spent* and Fe-SCR-*Spent*. The peak at 707.2 eV corresponded

to $2p_{3/2}$ of iron carbide. The peak at 720.2 eV was due to two overlapping components: the satellite $2p_{3/2}$ of α-Fe_2O_3 and $2p_{1/2}$ of iron carbide. The XPS analyses demonstrated that the Fe-Powder and Fe-SCR, after the pretreatments, showed similar Fe 2*p* regions.

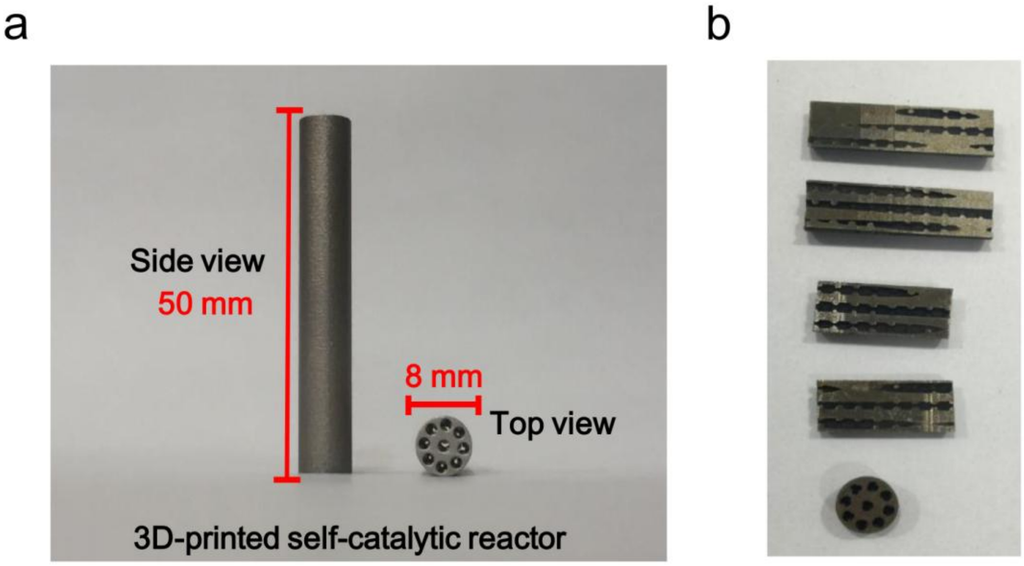

Supplementary Figure 18 | The Fe-SCR morphology after calcination process and FT synthesis. (**a**) The Fe-SCR morphology after calcination process, (**b**) the longitudinal section and cross-section of the Fe-SCR after FT synthesis.

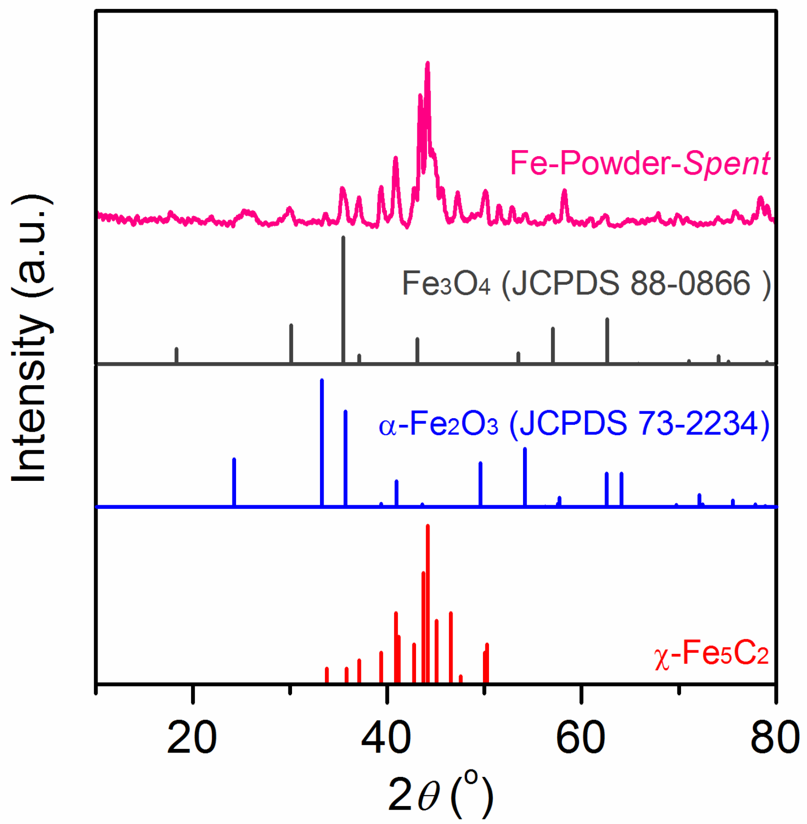

Supplementary Figure 19 | XRD patterns for the Fe-Powder-*Spent* after FT synthesis. The XRD results exhibited that χ-Fe_5C_2 was the main phase of Fe-Powder-*Spent*. The Fe_3O_4 and α-Fe_2O_3 were also observed, but their relative peak intensities were lower than that of χ-Fe_5C_2.

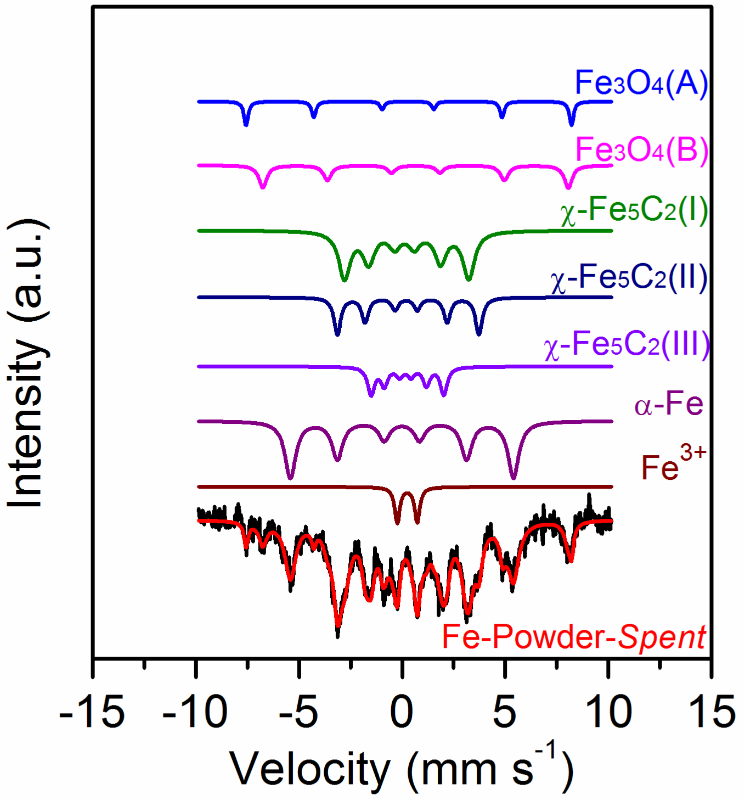

Supplementary Figure 20 | ^{57}Fe Mössbauer spectra of the Fe-Powder-*Spent*.[5,18] The phases of Fe^{3+}, α-Fe, χ-Fe_5C_2 and Fe_3O_4 were observed over the Fe-Powder-*Spent*. Although the iron phases were complex, the χ-Fe_5C_2 was still the main phase. This observation was in good agreement with the XRD analysis of Fe-Powder-*Spent*. The sextets with Hhf of 187, 213 and 109 kOe were attributed to three sites (I, II, and III) of the Hägg iron carbide (χ-Fe_5C_2). The sextets with Hhf of 490 and 460 kOe should correspond to tetrahedral A-sites and octahedral B-sites of the magnetite Fe_3O_4, respectively.

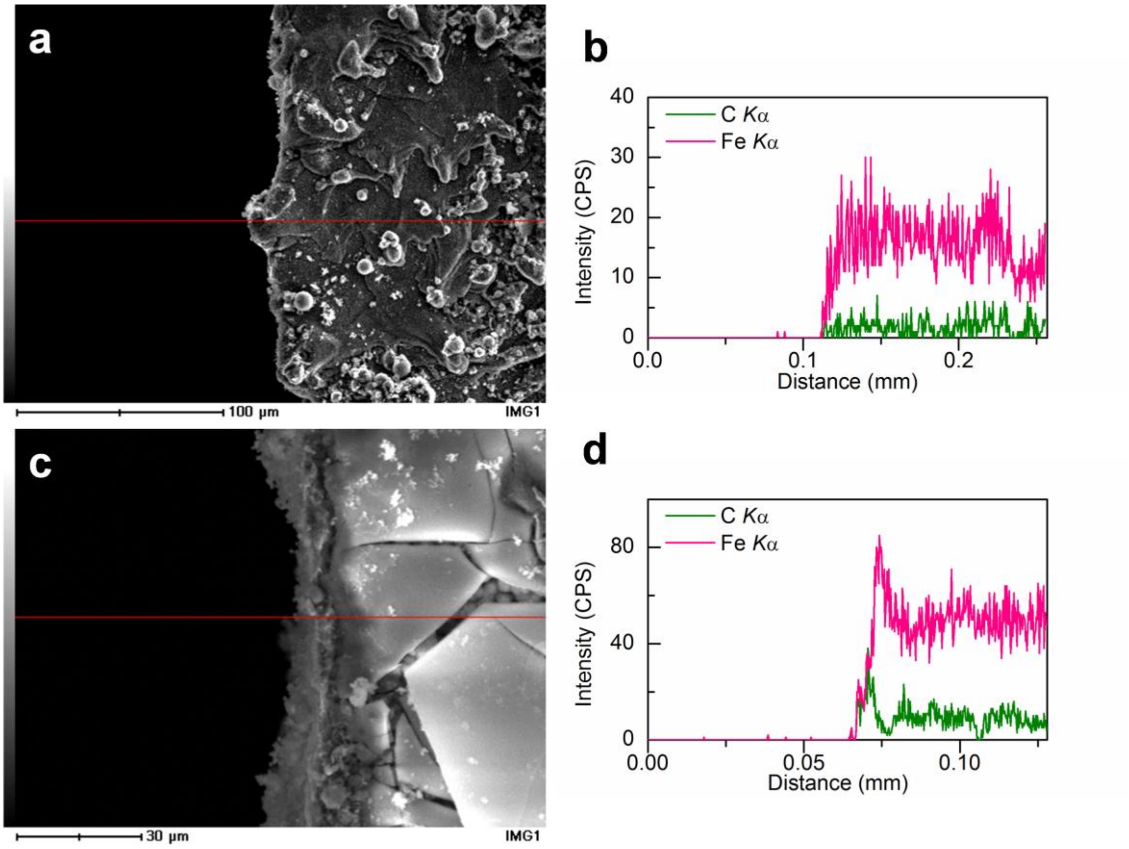

Supplementary Figure 21 | SEM images and EDS linear scan analyses for the cross-section of Fe-SCR and Fe-SCR-*Spent*. (**a**) SEM image for the cross-section of Fe-SCR. (**b**) EDS linear scan analysis for the cross-section of Fe-SCR. (**c**) SEM image for the cross-section of Fe-SCR-*Spent*. (**d**) EDS linear scan analysis for the cross-section of Fe-SCR-*Spent*.

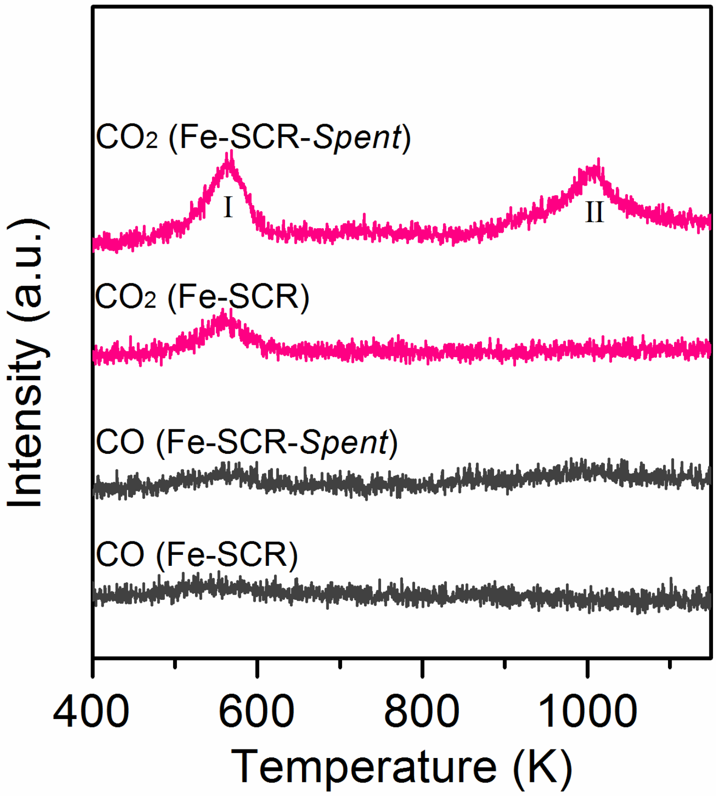

Supplementary Figure 22 | O_2-TPO profiles for the Fe-SCR and Fe-SCR-*Spent*.[19,20] In the O_2-TPO profiles, the peak I was observed on the Fe-SCR, indicating that inherent carbon species in the Fe-SCR were oxidized. In addition to the peak I, peak II was also observed on the Fe-SCR-*Spent*, suggesting that new carbon species were formed in the FT synthesis.

Supplementary Table 9 | O_2-TPO analysis for carbon content on the Fe-SCR and Fe-SCR-*Spent*.

Sample	Carbon from CO_2 peak I (μg)	Carbon from CO_2 peak II (μg)	Carbon from CO peak I (μg)	Carbon from CO peak II (μg)	Total carbon content in Fe-SCR (wt%)	Carbon retention from syngas (wt%)
Fe-SCR	77	/	5	/	0.01	/
Fe-SCR-*Spent*	110	141	8	3	0.03	0.14

The amount of carbon was calculated based on the O_2-TPO profiles. The analyses exhibited that the total carbon content on Fe-SCR and Fe-SCR-*Spent* was 0.01 wt% and 0.03 wt%, respectively. The carbon retention was 0.14 wt% of the carbon source of syngas.

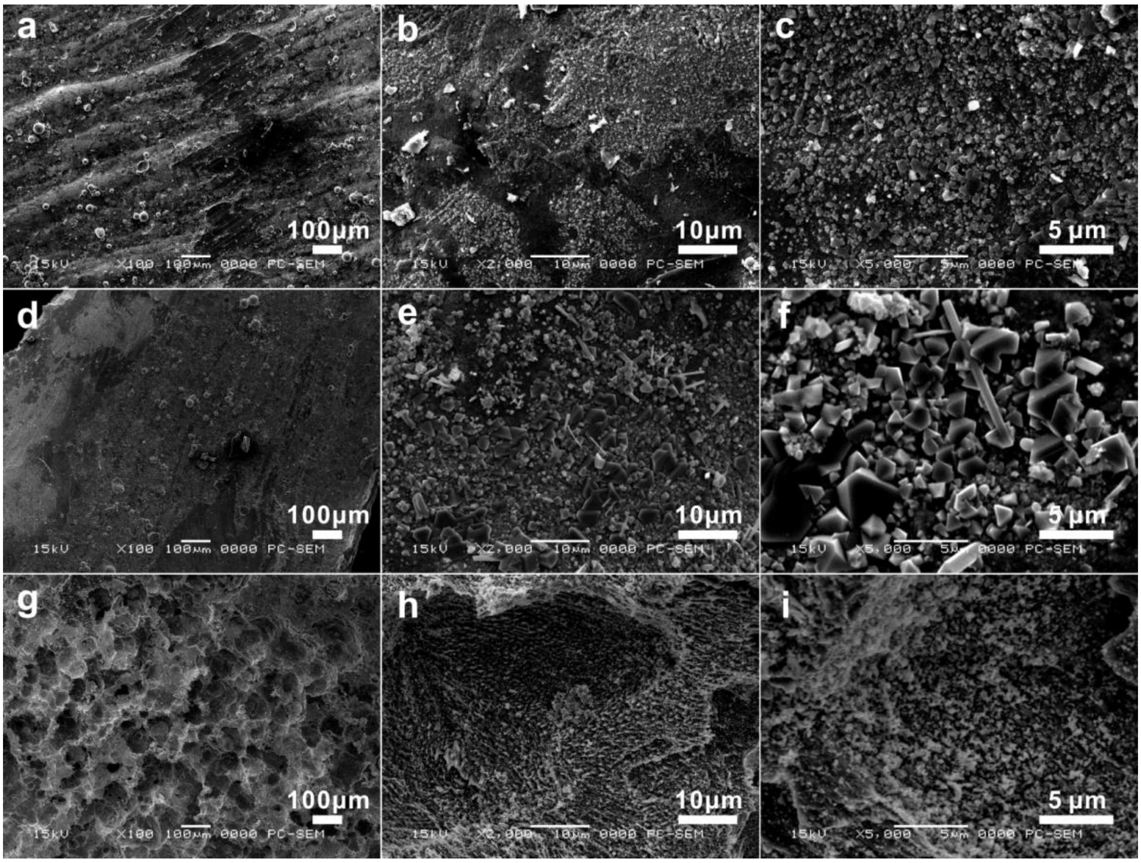

Supplementary Figure 23 | SEM images for inner surface of the Co-SCR. (**a**)-(**c**) The fresh Co-SCR; (**d**)-(**f**) the Co-SCR after calcination process; (**g**)-(**i**) the Co-SCR after acid treatment. The scale bar of 100 μm: (**a**), (**d**), and (**g**); the scale bar of 10 μm: (**b**), (**e**), and (**h**); the scale bar of 5 μm: (**c**), (**f**), and (**i**). The SEM results also displayed a grainy inner surface with a huge number of small particles on the surface.

Supplementary Table 10 | ICP analysis for metal elements in the liquid fuel.

Sample	Metal element (mg l^{-1})					
	Fe	Co	Cr	Mo	W	Ni
Liquid fuel (Fe-SCR)[a]	< DL[c]	/	< DL	< DL	/	< DL
Liquid fuel (Co-SCR)[b]	/	< DL	< DL	< DL	< DL	/

(**a**) Liquid fuel was obtained on the Fe-SCR after FT synthesis. (**b**) Liquid fuel was obtained on the Co-SCR after FT synthesis. (**c**) Detection limit was abbreviated as DL. In the ICP analysis, we tested four metal elements for each liquid-fuel sample. They were lower than the detection limit of ICP analysis. In addition, we further conducted XRF analysis on these two samples. The results also showed that the metal elements of Fe-SCR or Co-SCR did not contaminate the liquid fuel.

Supplementary Table 11 | Thermal conductivity of the Co-SCR and traditional FT catalyst supports.

Sample	Thermal conductivity ($W\ m^{-1}\ K^{-1}$)
Co-SCR	7
SiO_2	0.03
Al_2O_3	0.15

Thermal conductivity analysis was carried out on a portable thermal conductivity meter (TC3000E) by Xi' an Xiatech Electronics Co., Ltd. in China.

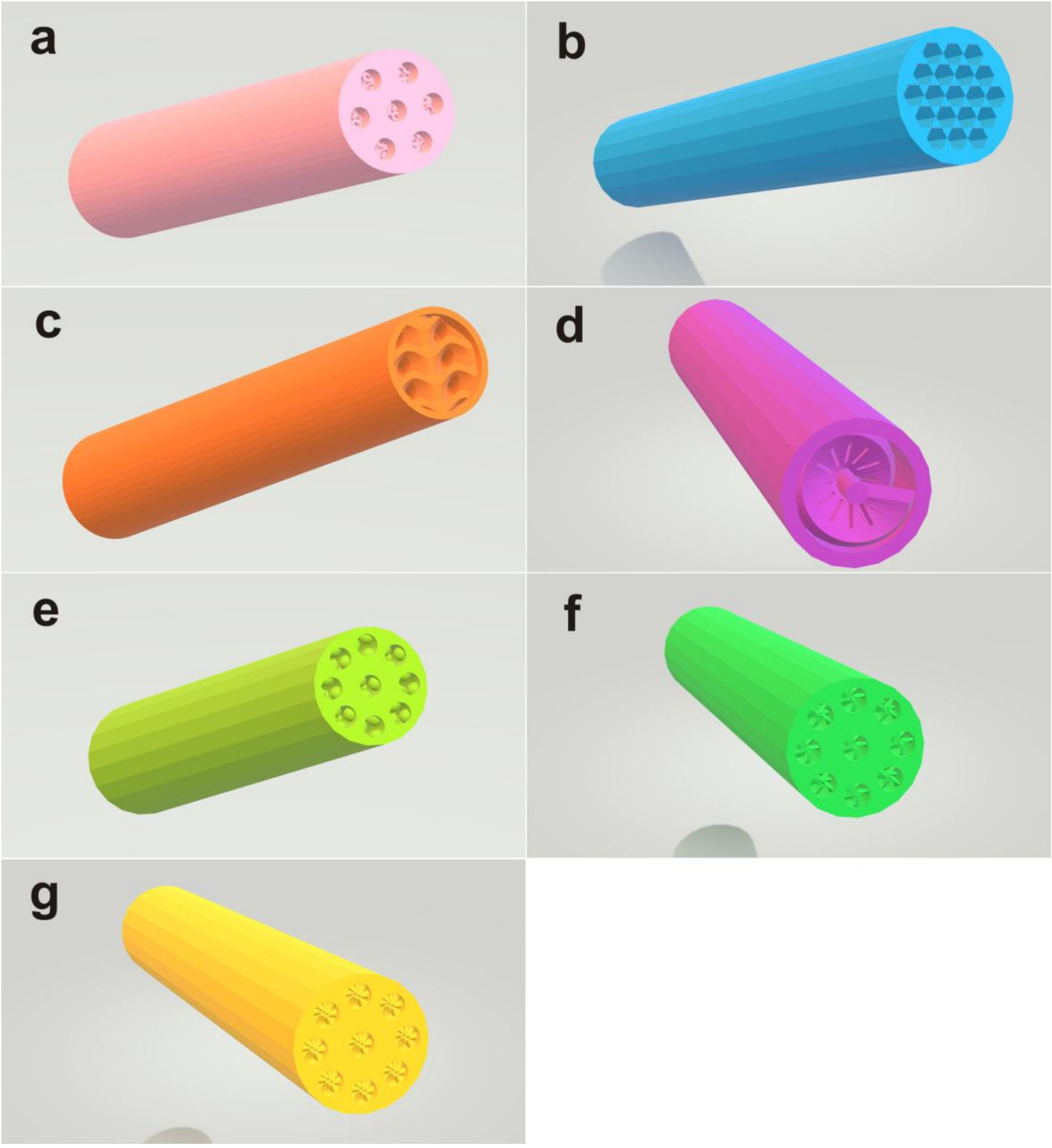

Supplementary Figure 24 | Geometrical structures of the Co-SCRs. (**a**) Co-SCR-1, (**b**) Co-SCR-2, (**c**) Co-SCR-3, (**d**) Co-SCR-4, (**e**) Co-SCR, (**f**) Co-SCR-5, (**g**) Co-SCR-6.

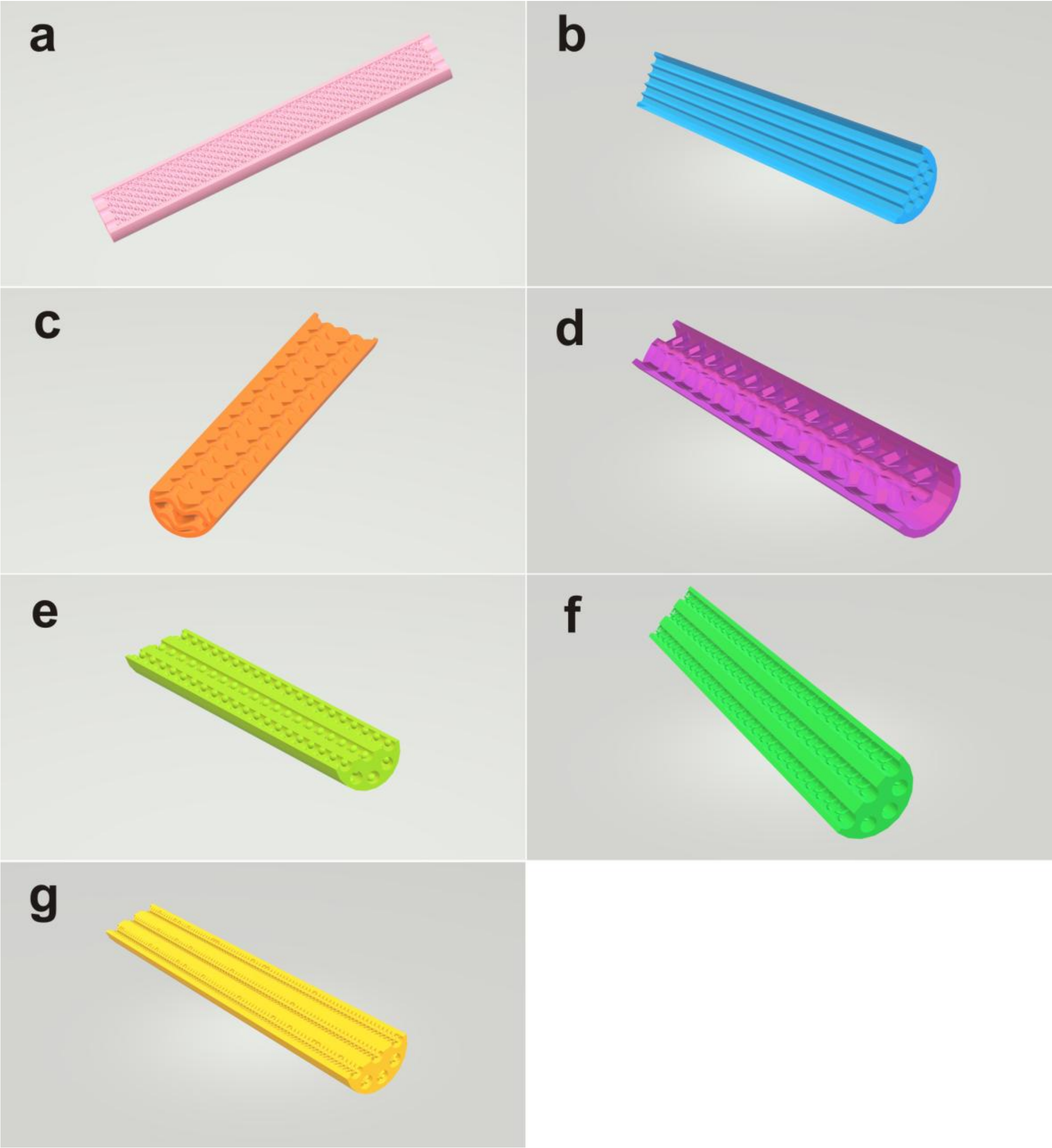

Supplementary Figure 25 | Longitudinal sections of the Co-SCRs. (**a**) Co-SCR-1, (**b**) Co-SCR-2, (**c**) Co-SCR-3, (**d**) Co-SCR-4, (**e**) Co-SCR, (**f**) Co-SCR-5, (**g**) Co-SCR-6.

Supplementary Table 12 | Inner surface, channel volume, passage time of syngas, and linear velocity of syngas for the Co-SCRs.[a]

Sample	Inner surface (10^{-3} m^2)	Channel volume (10^{-6} m^3)	Passage time[b] (s)	Linear velocity[c] (10^{-4} m s^{-1})
Co-SCR-1	3.4	1.3	3.9	1.0
Co-SCR-2	3.9	1.2	3.6	0.8
Co-SCR-3	1.8	0.5	1.5	1.9
Co-SCR-4	3.1	1.1	3.3	1.1
Co-SCR	3.9	0.6	1.8	0.8
Co-SCR-5	3.2	0.8	2.4	1.0
Co-SCR-6	7.7	0.4	1.2	0.4

(**a**) Internal surface and channel volume was obtained by CAD calculation (Rhinoceros 5.0). (**b**) The passage time was calculated according to the equation of $T_{passage} = V_{channel} / F_{CO+H2}$ ($T_{passage}$, $V_{channel}$ and F_{CO+H2} represent passage time, channel volume, and flow rate of syngas, respectively). (**c**) The linear velocity was calculated based on the equation of $V_{linear} = F_{CO+H2} / S_{cat.}$ (V_{linear}, F_{CO+H2} and $S_{cat.}$ represent linear velocity of syngas, flow rate of syngas and catalyst surface, respectively). Syngas conditions: temperature, 533 K; pressure, 2.0 MPa; flow rate, 20 ml min^{-1}.

Supplementary References

1. Li, J. *et al.* Integrated tuneable synthesis of liquid fuels via Fischer-Tropsch technology. *Nature Catalysis* **1,** 787–793 (2018).
2. Zhong, L. *et al.* Cobalt carbide nanoprisms for direct production of lower olefins from syngas. *Nature* **538,** 84–87 (2016).
3. Zheng,Y. *et al.* In situ X-ray diffraction study of reduction processes of Fe_3O_4- and $Fe_{1-x}O$-based ammonia-synthesis catalysts. *J. Solid State Chem.* **182,** 2385–2391 (2009).
4. Ghunaim, R. *et al.* $Fe_{1-x}Ni_x$ alloy nanoparticles encapsulated inside carbon nanotubes: controlled synthesis, structure and magnetic properties. *Nanomaterials* **8,** 576 (2018).
5. Wei, J. *et al.* Directly converting CO_2 into a gasoline fuel. *Nat. Commun.* **8,** 15174 (2017).
6. Chen, S. *et al.* Robust α-Fe_2O_3 nanorod arrays with optimized interstices as high-performance 3D anodes for high-rate lithium ion batteries. *J. Mater. Chem. A* **3,** 13377–13383 (2015).
7. Wojnarowska, A. *et al.* New mineralogical phases identified by Mössbauer measurements in Morasko meteorite. *Hyperfine Interact.* **186**, 167–171(2008).
8. Lyubutin, I. S. *et al.* Mössbauer spectroscopy and magnetic properties of hematite/magnetite nanocomposites. *J. Appl. Phys.* **106,** 034311 (2009).
9. Khollam, Y. B. *et al.* Microwave hydrothermal preparation of submicron-sized spherical magnetite (Fe_3O_4) powders. *Mater. Lett.* **56**, 571–577 (2002).
10. Mendili, Y. E. *et al.* New evidences of in situ laser irradiation effects on γ-Fe_2O_3 nanoparticles: a Raman spectroscopic study. *J. Raman Spectrosc.* **42,** 239–242 (2011).
11. de Faria, D. L., Venâncio Silva, A. S. & de Oliveira, M. T. Raman microspectroscopy of some iron oxides and oxyhydroxides. *J. Raman Spectrosc.* **28,** 873–878 (1997).
12. Shebanova, O. N. & Lazor, P. Raman study of magnetite (Fe_3O_4): laser-induced thermal effects and oxidation. *J. Raman Spectrosc.* **34,** 845–852 (2003).
13. Zhao, X. & Liu, X. A novel magnetic $NiFe_2O_4$@graphene-Pd multifunctional nanocomposite for practical catalytic application. *RSC Adv.* **5,** 79548–79555 (2015).

14. Jia, C.-J. *et al.* Large-scale synthesis of single-crystalline iron oxide magnetic nanorings. *J. Am. Chem. Soc.* **130**, 16968–16977 (2008).
15. Li, X. & Zhang, W. Sequestration of metal cations with zerovalent iron nanoparticless-A study with high resolution X-ray photoelectron spectroscopy (HR-XPS). *J. Phys. Chem. C* **111**, 6939-6946 (2007).
16. Butt, J. B. Carbide phases on iron-based Fischer-Tropsch synthesis catalysts part I: characterization studies. *Catal. Letters* **7**, 61–81(1990).
17. Yang, C. *et al.* Fe_5C_2 nanoparticles: a facile bromide-induced synthesis and as an active phase for Fischer-Tropsch synthesis. *J. Am. Chem. Soc.* **134,** 15814–15821 (2012).
18. Santos, V. P. *et al.* Metal organic framework-mediated synthesis of highly active and stable Fischer-Tropsch catalysts. *Nat. Commun.* **6,** 6451 (2015).
19. Zhang, J. *et al.* Synthesis of light olefins from CO hydrogenation over Fe-Mn catalysts: effect of carburization pretreatment. *Fuel* **109**, 116–123 (2013).
20. Hamilton, N. G. *et al.* The application of inelastic neutron scattering to investigate CO hydrogenation over an iron Fischer-Tropsch synthesis catalyst. *J. Catal.* **312**, 221–231 (2014).
