# Supplementary material for: Metal 3D printing technology for functional integration of catalytic system
Source: Nat Commun. 2020 Aug 14;11:4098. doi: 10.1038/s41467-020-17941-8 (PMC7428005; doi:10.1038/s41467-020-17941-8)
Supplement: Supplementary file 3 — Description of Additional Supplementary Files [file 41467_2020_17941_MOESM3_ESM.pdf]

Description of Additional Supplementary Files

File Name: Supplementary Movie 1
Description: Metal 3D printing processes via selective laser sintering: the alloy powder was first packed in a raw material vat and the base plate was mounted under the laser source; then, modeling data of the virtual reactors were set in the control system, and the reactors were rapidly printed on the base plate; after removing the residual alloy powder, the reactors were obtained by dismounting the base plate.
